# Anticoagulant effects of nafamostat administered via central venous catheter versus the ECMO circuit in adults receiving ECMO support

**DOI:** 10.3389/fcvm.2026.1876688

**Published:** 2026-09-23

**Authors:** Xinmei Chen, Kaixuan Wang, Xiaowen Wen, Qianqian Wang, Kun Chen, Hongjie Tong, Mudan Chen

**Affiliations:** 1 Department of Critical Care Medicine, Affiliated Jinhua Hospital Zhejiang University School of Medicine, Jinhua, China; 2 Department of Traditional Chinese Medicine, Affiliated Jinhua Hospital Zhejiang University School of Medicine, Jinhua, China; 3 Department of Critical Care Unit, The First Hospital of Jiaxing, Jiaxing, China; 4 Department of Critical Care Medicine, Pan’an County People's Hospital, Jinhua, China

**Keywords:** adults, anticoagulation, extracorporeal membrane oxygenation, infusion, nafamostat

## Abstract

**Objectives:**

To investigate the impact of administering nafamostat through central venous catheter versus extracorporeal membrane oxygenation (ECMO) circuit on anticoagulation efficacy during ECMO support.

**Materials and methods:**

This retrospective study included patients who received nafamostat for anticoagulation during ECMO support. Collected clinical data included age, sex, body weight, indication for ECMO, medical history, vital signs, laboratory test results, complications, and clinical outcomes. Patients were categorized into a central venous administration group and an ECMO-circuit administration group according to the site of nafamostat administration. Clinical parameters and outcomes were compared between the two groups.

**Results:**

A total of 102 patients were included in the analysis. The mean age was 51 ± 9 years, with 61 (59.8%) males and 41 (40.2%) females. There were no statistically significant differences between the two groups in age, body weight, sex distribution, ECMO indications, medical history, vital signs, laboratory findings, or acute physiology and chronic health evaluation II (APACHE II) scores (*P* > 0.05). The incidence of membrane oxygenator thrombosis was significantly higher in the central venous group than in the ECMO-circuit group (32.6% vs. 12.5%, *P* < 0.05). Central venous infusion of nafamostat was associated with an increased risk of membrane oxygenator thrombosis (OR:3.491, 95%CI 1.270–9.593, *P* < 0.05). The time required to achieve the anticoagulation target was significantly longer in the central venous group [4 (3–5) vs. 3 (3–4) hours, *P* < 0.05], and the mean daily dose of nafamostat was also significantly higher (1210 ± 261 vs. 1084 ± 132 mg, *P* < 0.05).

**Conclusions:**

Compared with central venous infusion, administration of nafamostat via the ECMO circuit was associated with a lower incidence of membrane oxygenator thrombosis, more rapid achievement of the anticoagulation target, and a lower daily dosage requirement. These findings are hypothesis-generating and require confirmation in prospective studies before informing clinical decisions regarding the route of nafamostat administration.

## 1 Introduction

Extracorporeal membrane oxygenation (ECMO) is a life-saving technique that provides temporary cardiac and pulmonary support for critically ill patients. With its increasing clinical application in recent years, a growing number of patients have benefited from this advanced life-support modality (1). However, like other extracorporeal life-support devices, ECMO is associated with significant complications. During ECMO support, exposure of blood to non-endothelial artificial surfaces activates the coagulation cascade, thereby increasing the risk of thrombus formation (2, 3). Consequently, systemic anticoagulation is essential throughout ECMO therapy.

Unfractionated heparin remains the most commonly used anticoagulant because of its availability, ease of use, and low cost. Nevertheless, heparin therapy is associated with an increased risk of bleeding and thrombocytopenia, particularly heparin induced thrombocytopenia (HIT), which has been linked to higher mortality rates (4). Therefore, safer and more effective anticoagulation strategies have long been sought. In recent years, nafamostat, a synthetic serine protease inhibitor, has emerged as a promising alternative anticoagulant. Previous studies suggest that nafamostat provides effective anticoagulation during ECMO support, especially in patients at high risk of bleeding (5, 6). However, due to its extremely short half-life of approximately 8 minutes, the pharmacodynamic effects of nafamostat may vary depending on the infusion site (7), potentially leading to differences in anticoagulant efficacy. Accordingly, this study compared central venous infusion and ECMO circuit infusion of nafamostat to evaluate differences in anticoagulant efficacy between the two administration routes, with the aim of providing evidence to guide the selection of the optimal infusion strategy during ECMO support.

## 2 Materials and methods

### 2.1 Study design and setting

This retrospective cohort study consecutively included patients who received nafamostat for anticoagulation during ECMO support at Affiliated Jinhua Hospital, Zhejiang University School of Medicine and The First Hospital of Jiaxing between January 2023 and September 2025. The study was approved by the Ethics Review Board of Affiliated Jinhua Hospital, Zhejiang University School of Medicine (Approval No. 2025-270) and The First Hospital of Jiaxing (Approval No. 2025-LP-025), and was conducted in accordance with the Declaration of Helsinki. Given the retrospective design, the requirement for informed consent was waived. This study was reported in accordance with the STROBE guidelines. The same anticoagulation protocol, nafamostat titration approach, and thrombosis surveillance procedure were followed at both participating centers throughout the study period.

### 2.2 Participants

Eligible participants were adult patients (≥18 years) who received ECMO support with nafamostat anticoagulation during the study period. Patients were excluded if more than 20% of their data were missing, if they had a history of hematologic disorders, gastrointestinal ulcers, or liver cirrhosis, or if they received continuous renal replacement therapy (CRRT) during ECMO support. For the remaining patients, missing data were minimal and a complete-case analysis was performed. No *a priori* power or sample-size calculation was performed; the cohort comprised all consecutive eligible patients treated during the predefined study period. This approach was consistent with the retrospective design of the study and enabled the inclusion of all consecutive patients who met the eligibility criteria, thereby minimizing selection bias.

### 2.3 Data collection and group allocation

Clinical data were collected retrospectively from electronic medical records and nursing documentation by two experienced attending physicians. The collected variables after ECMO initiation included vital signs, complete blood count, liver function tests, acute physiology and chronic health evaluation II (APACHE II) score, as well as sex, age, body weight, route of nafamostat administration, daily nafamostat dosage, time to achieve the anticoagulation target after infusion, ECMO duration, bleeding events, thrombotic events, hyperkalemia, blood product transfusion, length of hospital stay, ICU length of stay, and in-hospital survival. Complications occurring during ECMO support comprised bleeding events, thrombotic events, and hyperkalemia. Bleeding events included puncture-site bleeding, gastrointestinal bleeding, oronasal bleeding, and intracranial hemorrhage. Thrombotic events included membrane oxygenator thrombosis, deep vein thrombosis, intracardiac thrombosis, and ischemic stroke. During ECMO support, the membrane oxygenator was routinely inspected every 6 h by the responsible nurse to assess for thrombus formation. Hyperkalemia was defined as a serum potassium concentration >5.5 mmol/L on arterial blood gas analysis. Patients were followed until hospital discharge and were divided into a central venous administration group and an ECMO-circuit administration group according to the site of nafamostat administration. Relevant clinical outcomes and indicators were then compared between the two groups. Concomitant use of antiplatelet agents was also recorded; a proportion of patients in both groups received antiplatelet therapy, and the distribution was comparable between the two groups. No patient in this cohort underwent oxygenator or circuit exchange during the study period.

### 2.4 ECMO cannulation and maintenance

ECMO cannulation was performed percutaneously under ultrasound guidance by two or three experienced intensivists assisted by two nurses. A MAQUET (Germany) ECMO system was used in all cases. Venous cannulas with diameters of 21–26 Fr (MAQUET or Edwards) were inserted for drainage, and arterial (return) cannulas with diameters of 15–18 Fr (MAQUET, Edwards, or Medtronic) were used for reinfusion. For circuit priming, 20 mg of nafamostat was dissolved in 500 mL of normal saline. For maintenance anticoagulation, nafamostat (5 mg/mL) was initially administered at a rate of 7 mL/h under standard conditions, and the infusion rate was subsequently adjusted in 1 mL/h increments as clinically indicated. Activated partial thromboplastin time (APTT) was monitored every 2 hours, with the anticoagulation target defined as a post-membrane APTT of 60–100 seconds. The choice between central venous and ECMO-circuit administration was determined at the discretion of the treating clinician and was not standardized by protocol; when the ECMO-circuit route was used, nafamostat was infused pre-oxygenator through the same access port in all cases.

### 2.5 Outcomes

The primary outcome was the incidence of membrane oxygenator thrombosis. The secondary outcomes included the time to achieve the anticoagulation target, the mean daily dose of nafamostat, other bleeding complications (puncture-site, gastrointestinal, and oronasal bleeding, and intracranial hemorrhage), other thrombotic complications (deep vein thrombosis, intracardiac thrombosis, and ischemic stroke), hyperkalemia, ECMO duration, ICU length of stay, total hospital stay, transfusion requirements (red blood cell, platelet, and plasma), and in-hospital survival.

### 2.6 Statistical analysis

Statistical analysis was performed using R (version 4.4.3). Continuous variables with a normal distribution were expressed as mean ± standard deviation and comparisons between the two groups were conducted using independent samples t-test. If variances were unequal, the corrected t-test was applied. Continuous variables not following a normal distribution were presented as the median and interquartile range [M (Q1, Q3)] and comparisons between the two groups were conducted using the Mann–Whitney U test. Categorical variables were expressed as frequencies (percentages) and comparisons between groups were performed using the *χ*^2^ test. Multivariable logistic regression analysis was used to assess the factors associated with membrane oxygenator thrombosis. A broader set of candidate variables, including baseline demographic, clinical, and laboratory characteristics, was initially considered; covariates were then selected for the final multivariable model using a stepwise selection procedure, which retained route of administration, ECMO duration, and platelet count. The variance inflation factor (VIF) was calculated to assess multicollinearity among covariates, and no significant collinearity was identified (all VIF < 5). Time-to-event analysis for membrane oxygenator thrombosis was performed using the Kaplan–Meier method and compared using the log-rank test. A two-tailed P value < 0.05 was considered statistically significant.

## 3 Results

### 3.1 Study population and baseline characteristics

During the study period, 116 patients who received nafamostat for anticoagulation during ECMO support were identified from the medical records. Fourteen patients were excluded based on the predefined exclusion criteria, and a total of 102 patients were ultimately included in the final analysis (Figure 1). The mean age of the included patients was 51 ± 9 years, including 61 (59.8%) males and 41 (40.2%) females. Baseline demographic, clinical, and laboratory characteristics were comparable between the two groups (all *P* > 0.05; Table 1).

**Figure 1:**
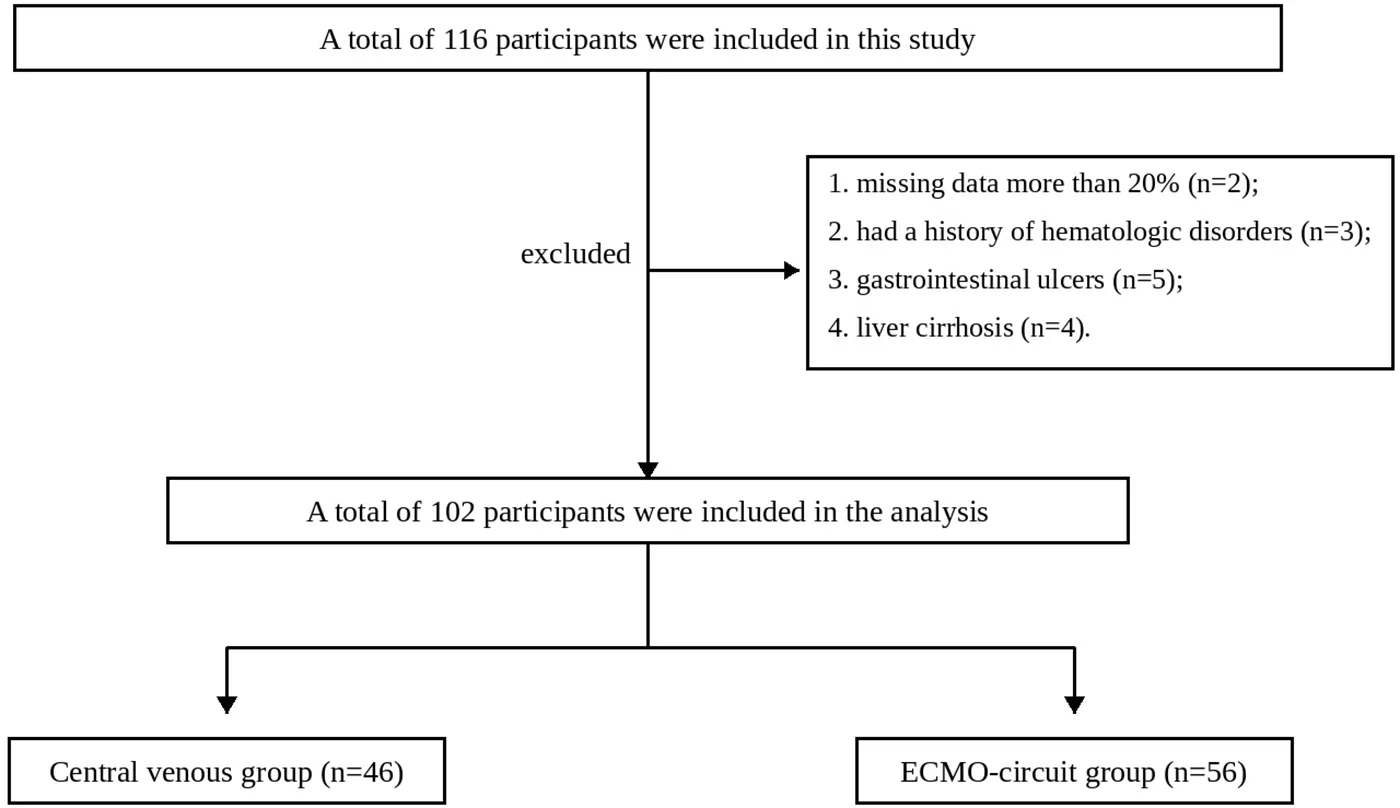
Flowchart of the patients' selection.

**Table 1:** Basic characteristics of patients.

Variables	Central venous group (*n* = 46)	ECMO-circuit group (*n* = 56)	*P* value
Age(years)	52 ± 9	50 ± 8	0.218
Weight(kg)	63.2 ± 6.7	63.8 ± 7.5	0.645
Sex, *n*(%)			
Male	27 (58.7%)	34 (60.7%)	0.836
Female	19 (41.3%)	22 (39.3%)	
ECMO modes, *n*(%)			1.000
VA	44 (95.7%)	54 (96.4%)	
VV	2 (4.3%)	2 (3.6%)	
Causes of ECMO, *n*(%)			
Cardiogenic shock	18 (39.1%)	26 (46.4%)	0.511
Cardiac arrest	19 (41.3%)	22 (39.3%)	0.836
Pulmonary embolism	7 (15.2%)	6 (10.7%)	0.497
Respiratory failure	2 (4.3%)	2 (3.6%)	0.841
Medical history, *n*(%)			
Hypertension	8 (17.4%)	5 (8.9%)	0.187
Diabetes	5 (10.9%)	10 (17.9%)	0.321
Coronary heart disease	10 (21.7%)	10 (17.9%)	0.623
Chronic kidney disease	3 (6.5%)	4 (7.1%)	0.902
COPD	3 (6.5%)	4 (7.1%)	0.902
Temperature(℃)	37.3 ± 0.6	37.4 ± 0.9	0.465
Respiration rate(/min)	16 ± 3	16 ± 2	0.241
Heart rate(/min)	94 ± 18	99 ± 20	0.173
Mean arterial pressure(mmHg)	72 ± 7	76 ± 6	0.119
White blood cells(10^9^/L)	10.5 ± 3.1	11.2 ± 2.9	0.491
Hemoglobin(g/L)	119 ± 14	113 ± 15	0.189
C-reactive protein(mg/L)	41.62 ± 19.3	38.22 ± 19.9	0.378
Platelets(10^9^/L)	209 ± 47	230 ± 44	0.155
Total bilirubin(*μ*mol/L)	17.8 ± 8.1	16.6 ± 7.6	0.643
ALT(U/L)	29.6 ± 14.2	31.2 ± 12.7	0.641
AST(U/L)	34.8 ± 8.0	37.6 ± 12.7	0.495
PT(s)	12.4 ± 4.0	11.1 ± 1.9	0.076
APTT(s)	42.3 ± 9.1	40.0 ± 9.3	0.218
INR	2.0 ± 0.3	2.0 ± 0.4	0.731
FDP (ug/mL)	96.91 ± 48.26	101.17 ± 45.39	0.648
D-dimer(ng/mL)	355 ± 165	348 ± 167	0.813
APACHE II	23 ± 7	24 ± 5	0.230

COPD, chronic obstructive pulmonary disease; AST, aspartate aminotransferase; ALT, alanine aminotransferase; PT, prothrombin time; APTT, activated partial thromboplastin time; INR, international normalized ratio; FDP, fibrinogen degradation products; APACHE II, acute physiology and chronic health.

### 3.2 Complications during anticoagulation and associated factors

Regarding the primary outcome, membrane oxygenator thrombosis occurred in 32.6% of patients in the central venous group, which was significantly higher than the 12.5% observed in the ECMO-circuit group (*P* < 0.05). Regarding bleeding events, the incidences of puncture site bleeding, gastrointestinal bleeding, and oronasal bleeding in the central venous group were 17.4%, 21.7%, and 13.0%, respectively, compared with 12.5%, 26.8%, and 16.1% in the ECMO-circuit group; there were no statistically significant differences in these bleeding complications between the two groups (all *P* > 0.05), and no intracranial hemorrhage occurred in either group. Regarding other thrombotic events, deep vein thrombosis occurred in 2 patients (4.3%) in the central venous group and 2 patients (3.6%) in the ECMO-circuit group (*P* > 0.05); no intracardiac thrombosis or ischemic stroke was observed in either group. The overall incidence of hyperkalemia in this cohort was 4.9%. At the patient level, at least one bleeding event of any type occurred in 12 patients (26.1%) in the central venous group and 18 patients (32.1%) in the ECMO-circuit group (*P* = 0.522), and at least one thrombotic event of any type occurred in 16 patients (34.8%) in the central venous group and 7 patients (12.5%) in the ECMO-circuit group (*P* = 0.009). Details of the complications are presented in Table 2. Multivariable logistic regression analysis demonstrated that the route of nafamostat administration was associated with membrane oxygenator thrombosis (Table 3).

**Table 2:** Complications during ECMO support, including bleeding events, thrombotic events, and hyperkalemia.

Variables	Central venous group (*n* = 46)	ECMO-circuit group (*n* = 56)	*P* value
Bleeding events, *n*(%)			
Puncture site bleeding	8 (17.4%)	7 (12.5%)	0.579
Gastrointestinal bleeding	10 (21.7%)	15 (26.8%)	0.555
Oronasal bleeding	6 (13.0%)	9 (16.1%)	0.782
Intracranial hemorrhage	0 (0.0%)	0 (0.0%)	>0.99
Thrombotic events, *n*(%)			
Membrane oxygenator thrombosis	15 (32.6%)	7 (12.5%)	0.014
Deep vein thrombosis	2 (4.3%)	2 (3.6%)	1.000
Intracardiac thrombosis	0 (0.0%)	0 (0.0%)	>0.99
Ischemic stroke	0 (0.0%)	0 (0.0%)	>0.99
Hyperkalemia, *n*(%)	2 (4.3%)	3 (5.4%)	0.860

Hyperkalemia was defined as a serum potassium concentration >5.5 mmol/L on arterial blood gas analysis. Data are presented as *n* (%).

**Table 3:** Multivariable regression analysis of the factors associated with membrane oxygenator thrombosis.

Variables	OR	95%CI	*P* value
Route of NM administration	3.491	1.270–9.593	0.015
ECMO duration	1.031	0.888–1.198	0.686
Platelets	0.995	0.984–1.007	0.421

NM, nafamostat; ECMO, extracorporeal membrane oxygenation.

### 3.3 Clinical outcomes

The time required to achieve the anticoagulation target was significantly longer in the central venous group [4 (3–5) hours] than in the ECMO-circuit group [3 (3–4) hours] (*P* < 0.05). In addition, the mean daily dose of nafamostat was significantly higher in the central venous group (1,210 ± 261 mg) compared with the ECMO-circuit group (1,084 ± 132 mg) (*P* < 0.05). In contrast, there were no statistically significant differences between the two groups in ECMO duration, in-hospital survival, ICU length of stay, or total hospital stay (all *P* > 0.05). Total platelet transfusion did not reach statistical significance (*P* = 0.056). Detailed outcome data are presented in Table 4. Kaplan–Meier curves for the primary outcome, membrane oxygenator thrombosis, are shown in Figure 2; the cumulative incidence was significantly higher in the central venous group than in the ECMO-circuit group (log-rank *P* = 0.015), consistent with the results of the multivariable analysis (Table 3).

**Table 4:** Clinical outcomes.

Variables	Central venous group (*n* = 46)	ECMO-circuit group (*n* = 56)	*P* value
Time to anticoagulation target(h)	4 (3, 5)	3 (3, 4)	＜0.01
NM dose per day (mg)	1,210 ± 261	1,084 ± 132	0.02
ECMO duration(days)	11 (9, 15)	13 (10, 15)	0.415
In-hospital survival, n(%)	24 (52.2%)	26 (46.4%)	0.564
Length of ICU stay(days)	17 (11, 23)	20 (16, 24)	0.362
Hospitalization duration(days)	19 (11, 30)	23 (18, 27)	0.073
Total red cell transfusion(U)	8.0 (5.5, 10.0)	8.0 (6.0, 10.0)	0.898
Total platelet transfusion(U)	15 (10, 20)	29 (20, 30)	0.056
Total plasma transfusion(mL)	400 (225, 613)	550 (363, 750)	0.090

NM, nafamostat.

**Figure 2:**
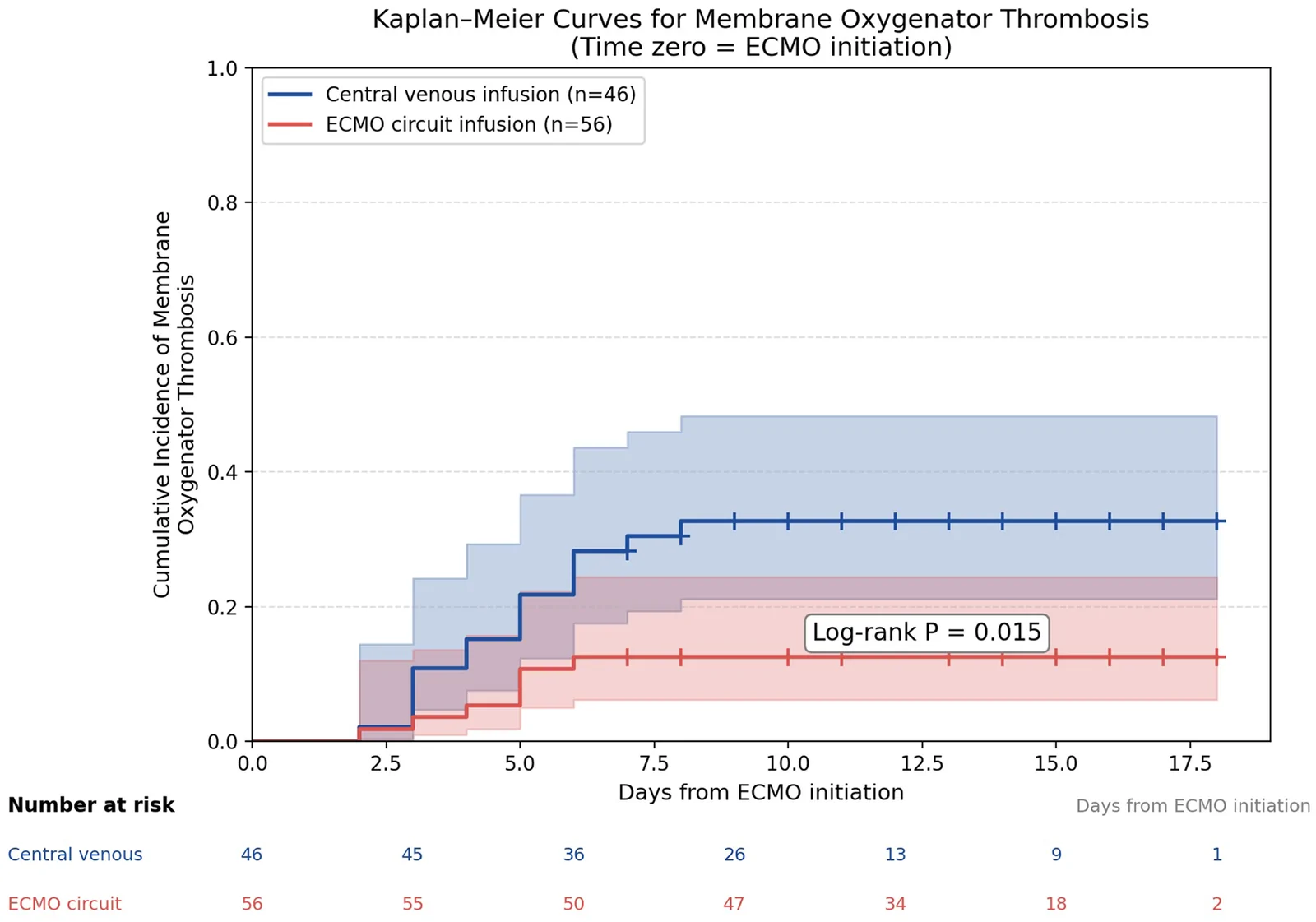
Kaplan–Meier curves for the cumulative incidence of membrane oxygenator thrombosis.

## 4 Discussion

This study suggests that, compared with central venous infusion, administration of nafamostat via the ECMO circuit is associated with more favorable anticoagulation profiles during ECMO support, including more rapid achievement of the anticoagulation target, lower daily drug requirements, and a lower incidence of membrane oxygenator thrombosis. As a retrospective, hypothesis-generating analysis, these findings represent associations rather than causal relationships.

Our findings suggest that the administration route may influence anticoagulant exposure within the ECMO circuit. Central venous infusion was associated with a higher incidence of membrane oxygenator thrombosis in our cohort. This association may reflect lower local nafamostat exposure around the oxygenator, a thrombosis-prone component of the circuit, although drug concentrations were not measured in the present study. Lower circuit exposure could also explain the longer time to achieve the anticoagulation target and the higher daily nafamostat requirement observed with central venous infusion.

Previous studies have reported heterogeneous clinical outcomes with nafamostat during ECMO support. In a retrospective propensity score-matched study of patients receiving VA-ECMO, Lim et al. found no significant difference in thromboembolic events between nafamostat and heparin, whereas bleeding was significantly more frequent in the nafamostat group in both the unmatched and matched cohorts (*P* = 0.03), with nafamostat use being the only significant risk factor for bleeding on multivariate analysis (hazard ratio 2.4, 95% CI 1.07–5.36, *P* = 0.032) (8). A systematic review of 11 retrospective studies likewise reported conflicting findings for bleeding and thrombosis, with substantial heterogeneity in nafamostat dose and anticoagulation-monitoring strategies (5). A more recent systematic review and meta-analysis of 2,775 adult ECMO patients comparing unfractionated heparin, direct thrombin inhibitors, low-molecular-weight heparin, nafamostat, and no anticoagulation found that direct thrombin inhibitors and low-molecular-weight heparin had a more favorable overall efficacy and safety profile, while noting that evidence specific to nafamostat remained limited by the small number of available studies and patients (9). Consistent with this, comparative studies of heparin versus direct thrombin inhibitors (10) and bivalirudin (11) during ECMO, as well as a network meta-analysis of anticoagulation strategies in this setting (12), have similarly highlighted the heterogeneity of outcomes across agents and the lack of consensus on an optimal regimen. These observations suggest that reported outcomes may be influenced by patient characteristics, ECMO mode, treatment selection, and monitoring protocols. Unlike those studies, which compared different anticoagulants, the present study compared two administration routes of nafamostat; therefore, our findings should be interpreted as route-related differences in circuit anticoagulation rather than as evidence of superiority of nafamostat over heparin. In addition, a prospective PK/PD study reported distinct pharmacodynamic profiles in central venous and ECMO-circuit samples from patients receiving pre-pump nafamostat infusion, indicating that systemic and circuit-side measurements may not reflect anticoagulant effects equivalently. Although that study did not compare different infusion routes, its findings provide indirect mechanistic support for the possibility that local circuit exposure is relevant to anticoagulation during ECMO (7). Advances in bioanalytical methods, such as LC-MS/MS-based quantification of nafamostat (13), together with broader clinical experience with nafamostat mesylate outside the ECMO setting (14), may further facilitate future pharmacokinetic studies in this population.

This study has several limitations. First, its retrospective design inherently carries a risk of selection bias, and the small sample size may limit the statistical power of our findings. Furthermore, data were collected from two centers without adjusting for potential center effects. Although ECMO mode (VA vs. VV) is a recognized determinant of mortality, its distribution was nearly identical between the two infusion-route groups in this cohort (VV: 2/56 vs. 2/46), and only 4 of 102 patients received VV-ECMO overall. Given this sparse and balanced distribution, ECMO mode was not included as a matching variable in a propensity score model, as such a small subgroup would not support stable estimation. We therefore consider it unlikely to have materially confounded the comparison of thrombotic outcomes between groups, although we acknowledge that a larger, prospectively designed cohort with adequate representation of both ECMO modes would allow for more rigorous propensity-score-based adjustment. Second, the route of nafamostat administration was not standardized but was determined at the discretion of the treating clinician, which may have introduced variability. Additionally, the cumulative dose of nafamostat prior to the occurrence of thrombosis was not comprehensively recorded. Third, the assessment of thrombotic endpoints relied primarily on nursing observation and documentation without blinding, which introduces a potential risk of detection bias. Fourth, we did not routinely monitor plasma free hemoglobin levels to assess hemolysis, which is a relevant complication during ECMO support. Fifth, no *a priori* power calculation was performed. Sixth, the Kaplan–Meier analysis of membrane oxygenator thrombosis did not account for competing risks (e.g., death or ECMO discontinuation before thrombosis could occur), which may affect the interpretation of the cumulative incidence estimates. Seventh, new pulmonary embolism occurring after ECMO initiation was not systematically tracked as an outcome. Eighth, circuit thrombosis at sites other than the membrane oxygenator, such as the pump head or tubing, was not systematically assessed. Ninth, oxygenator lifespan was not reported as an independent metric; ECMO duration was used as an approximate surrogate, though this may not fully capture oxygenator-specific performance. Tenth, because patients who received CRRT during ECMO support were excluded from this cohort, our findings do not address anticoagulation strategies in that overlapping clinical scenario; nafamostat has been separately evaluated as an anticoagulant during CRRT (15, 16), alongside regional citrate anticoagulation (16) and anticoagulant-free strategies in patients at high bleeding risk (17), and these approaches warrant separate consideration outside the ECMO context. Finally, potential differences between pre-pump and post-pump nafamostat infusion could not be evaluated due to the lack of relevant data, and this issue warrants further investigation. Future studies should ideally take the form of a prospective, protocolized comparison incorporating dual-site pharmacokinetic sampling, which would allow direct evaluation of circuit-level drug exposure and provide stronger evidence for causal inference than the present retrospective comparison.

## 5 Conclusion

In conclusion, in this retrospective cohort, administration of nafamostat via the ECMO circuit was associated with more rapid achievement of the anticoagulation target, lower daily drug dosages, and fewer membrane oxygenator thrombotic complications compared with central venous infusion. These associations are hypothesis-generating rather than causal and should not be used to guide the choice of nafamostat administration route in clinical practice. Confirmation in a prospective, protocolized study with dual-site pharmacokinetic sampling is needed before any causal or practice-changing conclusions can be drawn.

## Data Availability

The original contributions presented in the study are included in the article/Supplementary Material, further inquiries can be directed to the corresponding authors.
